# Continuous saline bladder irrigation for two hours following transurethral resection of bladder tumors in patients with non-muscle invasive bladder cancer does not prevent recurrence or progression compared with intravesical Mitomycin-C

**DOI:** 10.1186/s12894-018-0408-6

**Published:** 2018-10-24

**Authors:** Andrew T. Lenis, Kian Asanad, Maher Blaibel, Nicholas M. Donin, Karim Chamie

**Affiliations:** 1 David Geffen School of Medicine at the University of California Los Angeles, 300 Stein Plaza, Suite 348, Los Angeles, California 90095 USA; 2 Department of Urology, Health Services Research Group, David Geffen School of Medicine at UCLA, Los Angeles, California USA; 3 Jonsson Comprehensive Cancer Center, David Geffen School of Medicine at UCLA, Los Angeles, California USA; 4 Riverside School of Medicine, University of California, Riverside, California USA

**Keywords:** Bladder cancer, Therapeutic irrigation, Mitomycin-C, Recurrence, Outcome assessment

## Abstract

**Background:**

Intravesical Mitomycin-C (MMC) following transurethral resection of bladder tumor (TURBT), while efficacious, is associated with side effects and poor utilization. Continuous saline bladder irrigation (CSBI) has been examined as an alternative. In this study we sought to compare the rates of recurrence and/or progression in patients with NMIBC who were treated with either MMC or CSBI after TURBT.

**Methods:**

We retrospectively reviewed records of patients with NMIBC at our institution in 2012–2015. Perioperative use of MMC (40 mg in 20 mL), CSBI (two hours), or neither were recorded. Primary outcome was time to recurrence or progression. Descriptive statistics, chi-squared analysis, Kaplan-Meier survival analysis, and Cox multivariable regression analyses were performed.

**Results:**

205 patients met inclusion criteria. Forty-five (22.0%) patients received CSBI, 71 (34.6%) received MMC, and 89 (43.4%) received no perioperative therapy. On survival analysis, MMC was associated with improved DFS compared with CSBI (*p* = 0.001) and no treatment (*p* = 0.0009). On multivariable analysis, high risk disease was associated with increased risk of recurrence or progression (HR 2.77, 95% CI: 1.28–6.01), whereas adjuvant therapy (HR 0.35, 95% CI: 0.20–0.59) and MMC (HR 0.43, 95% CI: 0.25–0.75) were associated with decreased risk.

**Conclusions:**

Postoperative MMC was associated with improved DFS compared with CSBI and no treatment. The DFS benefit seen with CSBI in other studies may be limited to patients receiving prolonged irrigation. New intravesical agents being evaluated may consider saline as a control given our data demonstrating that short-term CSBI is not superior to TURBT alone.

## Background

Non-muscle invasive bladder cancer (NMIBC) accounts for approximately 70% of new cases of urothelial carcinoma of the bladder. [1] NMIBC has been considered a chronic disease due to its high risk of future complications, including recurrence, which necessitates frequent monitoring and surveillance. The lifelong risk of recurrence and repeated interventions contributes to poor physician and patient compliance with published guidelines, and it significantly burdens the healthcare system from a financial standpoint. [2, 3] Therefore, strategies to prevent recurrence and future complications are paramount to reducing long-term morbidity and mortality.

The standard adjuvant therapy following transurethral resection of bladder tumor (TURBT) for NMIBC is intravesical instillation of Mitomycin-C (MMC), which has been shown to decrease rates of recurrence by approximately 11%, although this is variable depending on the number of and time from prior recurrences. [4, 5] The posited mechanism of action is to prevent free-floating tumor cells in the urine following TURBT from re-implanting onto the bladder wall. Although rare, MMC can potentially cause several significant side effects, including severe lower urinary tract symptoms, persistent chronic bladder pain, and even bladder necrosis in case reports. [6] Furthermore, MMC is contraindicated when there is a concern for bladder perforation and when there is significant post-operative gross hematuria. Considering these limitations, there is an urgent need for alternative strategies to prevent the re-implantation of tumor cells following TURBT, to reduce recurrence and minimize the morbidity of the disease. A 2012 Cochrane review of intravesical gemcitabine yielded conflicting results. [7] Apaziquone is a novel intravesical alkylating agent that has demonstrated safety and tolerability in patients as a post-TURBT instillation and is being evaluated in Phase 3 clinical trials (NCT02563561). [8] Alternatively, several groups have utilized sterile water and saline irrigation over 18–24 h as a strategy to lyse floating tumors cells and prevent the re-implantation of cells into the bladder wall. [9, 10] In our current study, we sought to evaluate continuous bladder irrigation with isotonic (0.9% NaCl) normal saline (CBSI) for two hours following TURBT as a strategy to reduce recurrence or progression in patients with NMIBC.

## Methods

### Patient cohort

Patients undergoing endoscopic resection of bladder tumors at our institution between March 2012 and July 2015 were identified from the medical record by Current Procedure Terminology (CPT)-4 codes for transurethral biopsy and resection (52204, 52214, 52224, 52234, 52235, 52240). Pathologic and clinical reports were reviewed, and patients with NMIBC were selected for inclusion in the cohort. We excluded all patients with variant histology, including small cell, squamous cell, adenocarcinoma, lymphepithelioid, sarcomatoid, and micropapillary disease. We also excluded patients with a diagnosis of upper tract urothelial carcinoma within one year, unresectable volume of tumor, known metastatic disease, less than three months of follow-up, or patients who underwent cystectomy within three months of diagnosis. Patients were categorized based on a modified AUA Risk Stratification for NMIBC. [11] Low risk was defined as a solitary LG lesion < 2 cm. Intermediate risk was defined as any LG T1, solitary LG Ta > 2 cm, multiple LG Ta, solitary HG Ta < 2 cm, or a history of LG NMIBC. High risk was defined as any CIS, HG T1, HG Ta > 2 cm, multiple HG Ta, or any history of HG Ta lesions or BCG recurrence. Modification of the AUA risk groups was made in order to conform to the size criteria used in the current procedural terminology codes for TURBT. Follow-up was calculated based on the time of the last cystoscopy. All study conduct was approved by the Institutional Review Board at our institution.

### Independent variables

All patients received adjuvant CSBI, adjuvant MMC, or no adjuvant treatment at the discretion of the operating surgeon. Typically, patients for whom there was a concern for bladder perforation were not given CSBI or MMC. MMC was given as an instillation of 40 mg in 20 mL of saline. Following a dwell time of 60–90 min, the MMC was drained from the bladder and the catheter was left in place if deemed necessary by the surgeon. CSBI was performed by placement of a three-way Foley catheter at the conclusion of the case and was left running for approximately two hours post-operatively. The rate was kept at maximum flow without titration for this time. Patients did not require an overnight stay specifically for CSBI.

### Dependent variables

Our dependent variable of interest was time to recurrence or progression. Recurrence was defined as the presence of pathologically confirmed urothelial carcinoma on biopsy or repeat resection. Patients who were found to have a lesion visible on cystoscopy that warranted intervention in the office (e.g. fulguration) were also classified as having disease recurrence. Cytology results obtained at the time of office fulguration were recorded. Progression was defined as any increase in grade or stage of disease.

### Statistical analysis

Comparisons between categorical variables were tested using Chi-squared analysis and Fisher’s exact test when appropriate. The two-sample Student’s t-test was used to test for differences between continuous variables. Differences in disease-free survival (DFS) were analyzed using the Kaplan-Meier method. Cox proportional hazards models were used to estimate hazards ratios for covariates of interest. All statistical analyses were performed with Stata statistical software version 14 (StataCorp, College Station, TX).

## Results

A total of 205 patients underwent TURBT for NMIBC during the study period and met all inclusion criteria. Mean age was 71.9 (SD = 11.4) years and 81.5% were male. Low grade (LG) and high grade (HG) were the primary grades in 105 (51.2%) and 100 (48.8%) patients, respectively. Stage was Ta without CIS, Ta with CIS, T1 without CIS, T1 with CIS, and CIS alone in 126 (61.5%), 12 (5.9%), 36 (17.6%), 13 (6.3%), and 18 (8.8%) patients, respectively. Tumor size was < 0.5 cm, 0.5–2 cm, 2–5 cm, and > 5 cm in 20 (9.8%), 90 (43.9%), 45 (21.9%), and 50 (24.4%) patients, respectively. Multiple tumors were present in 105 (51.2%) patients and 75 (36.6%) had a history of NMIBC. A modified AUA risk stratification as discussed in the methods resulted in 23 (11.2%) low risk patients, 80 (39%) intermediate risk patients, and 102 (49.8%) high risk patients. As immediate perioperative therapy, a total of 45 (22.0%) patients had CSBI, 71 (34.6%) had MMC, and 89 (43.4%) had no perioperative therapy. Only 36 (19.8%) of patients with intermediate or high risk disease underwent a restaging TURBT. Eighty-six (42.0%) patients received adjuvant intravesical therapy, most commonly with bacillus Calmette-Guérin (BCG *n* = 76), BCG + interferon (*n* = 6), Gemcitabine (*n* = 2), or MMC (n = 2). Table 1 and Table 2 summarize the cohort characteristics stratified by perioperative treatment and recurrence and progression, respectively.

**Table 1:** Cohort characteristics stratified by perioperative treatment

Variable	No treatment	MMC	CSBI	*p*-value
Total no. of patients	89	71	45	–
Age, mean (SD)	73.2 (11.2)	68.2 (12.3)	75.3 (8.9)	< 0.002+
Gender, *n* (%)				0.54
Male	75 (84.3)	55 (77.5)	37 (83.2)	
Female	14 (15.7)	16 (22.5)	8 (17.8)	
Grade, *n* (%)				0.9
High	45 (50.6)	34 (47.9)	21 (46.7)	
Low	44 (49.4)	37 (52.1)	24 (53.3)	
Stage, *n* (%)				0.03*
Ta without CIS	55 (61.8)	41 (57.8)	30 (66.7)	
Ta with CIS	3 (3.4)	4 (5.6)	5 (11.1)	
T1 without CIS	13 (14.6)	18 (25.4)	5 (11.1)	
T1 with CIS	4 (4.5)	6 (8.5)	3 (6.7)	
CIS only	14 (15.7)	2 (2.8)	2 (4.4)	
Tumor size, *n* (%)				0.12*
< 0.5 cm	11 (12.36)	3 (4.2)	6 (13.3)	
0.5–2.0 cm	33 (37.1)	41 (57.8)	16 (35.6)	
2.0–5.0 cm	22 (24.7)	13 (18.3)	10 (22.2)	
> 5.0 cm	23 (25.8)	14 (19.7)	13 (28.9)	
Multiple tumors, *n* (%)	47 (52.8)	36 (50.7)	22 (48.9)	0.91
Recurrent disease, *n* (%)	40 (45.0)	23 (32.4)	12 (26.7)	0.08
AUA Risk Stratification				0.72
Low risk	10 (11.2)	6 (8.5)	7 (15.6)	
Intermediate risk	34 (38.2)	31 (43.7)	15 (33.3)	
High risk	45 (50.6)	34 (47.9)	23 (51.1)	
Restaging resection, *n* (%)	8 (9.0)	18 (25.4)	10 (22.2)	0.02
Adjuvant therapy, *n* (%)	35 (39.3)	35 (49.3)	16 (35.6)	0.28
Follow-up in months, median [IQR]	14 [6–28]	23 [11–32]	13 [9–19]	< 0.01§

*MMC* Mitomycin-C, *CSBI* continuous saline bladder irrigation, *SD* standard deviation, *CIS* carcinoma in situ. +One-way ANOVA. *Fisher’s exact test. §non-parametric equality of medians test

**Table 2:** Cohort characteristics stratified by Recurrence or Progression

Variable	Recurrence or Progression	No Recurrence or Progression	p-value
Total no. of patients	90	115	–
Age, mean (SD)	73.6 (10.8)	70.6 (11.8)	0.07+
Gender, *n* (%)			0.81
Male	74 (82.2)	93 (80.9)	
Female	16 (17.8)	22 (19.1)	
Grade, *n* (%)			0.38
High	47 (52.2)	53 (46.1)	
Low	43 (47.8)	62 (53.9)	
Stage, *n* (%)			0.09*
Ta without CIS	55 (61.1)	71 (61.7)	
Ta with CIS	3 (3.3)	9 (7.8)	
T1 without CIS	14 (15.6)	22 (19.1)	
T1 with CIS	5 (5.6)	8 (7.0)	
CIS	13 (14.4)	5 (4.4)	
Tumor size, *n* (%)			0.09
< 0.5 cm	14 (15.6)	6 (5.2)	
0.5–2.0 cm	37 (41.1)	53 (46.1)	
2.0–5.0 cm	17 (18.9)	28 (24.4)	
> 5.0 cm	22 (24.4)	28 (24.3)	
Multiplicity of tumor, *n* (%)	56 (62.2)	49 (42.6)	< 0.01
Recurrent disease, *n* (%)	42 (46.7)	33 (28.7)	< 0.01
AUA Risk Stratification			0.07
Low risk	9 (10.0)	14 (12.2)	
Intermediate risk	28 (31.1)	52 (45.2)	
High risk	53 (58.9)	49 (42.6)	
Restaging resection, *n* (%)	12 (13.3)	24 (20.9)	0.16
Adjuvant therapy, *n* (%)	32 (35.6)	54 (47.0)	0.10
Perioperative treatment, *n* (%)			0.004
None	47 (52.2)	42 (36.5)	
MMC	20 (22.2)	51 (44.4)	
CSBI	23 (25.6)	22 (19.1)	

*MMC* Mitomycin-C, *CSBI* continuous saline bladder irrigation, *SD* standard deviation, *CIS* carcinoma in situ. +One-way ANOVA. *Fisher’s exact test

**Fig. 1:**
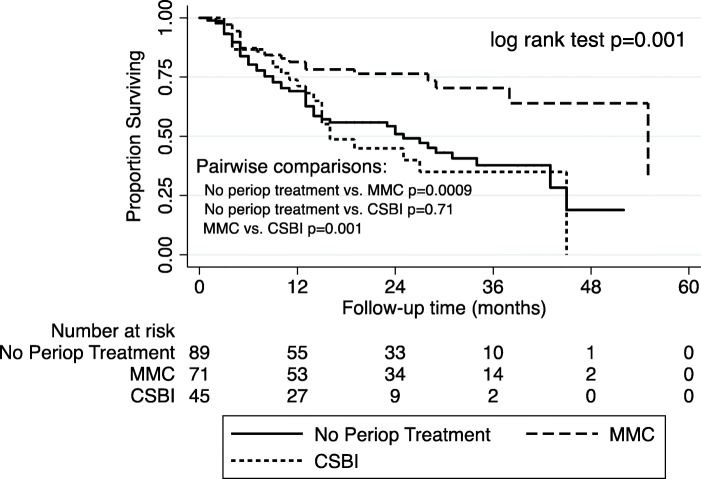
“DFS in Patients with NMIBC”. Kaplan-Meier survival curve for all patients with NMIBC stratified by perioperative treatment. MMC, Mitomycin-C. CSBI, continuous saline bladder irrigation

**Fig. 2:**
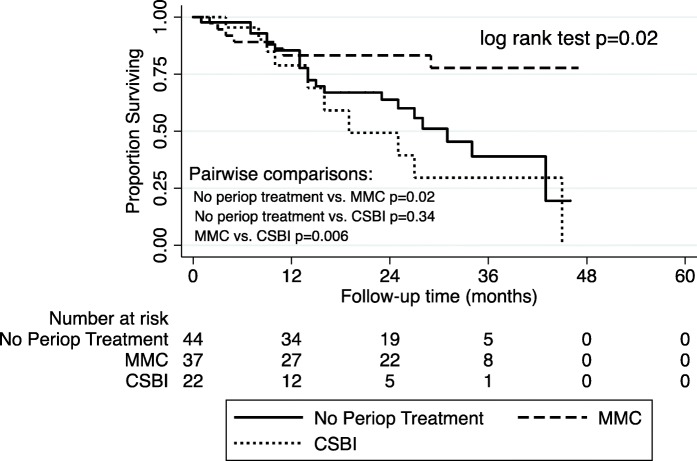
“DFS in Patients with Low and Intermediate Risk”. Kaplan-Meier survival curve for patients with low and intermediate risk disease stratified by perioperative treatment. MMC, Mitomycin-C. CSBI, continuous saline bladder irrigation

**Fig. 3:**
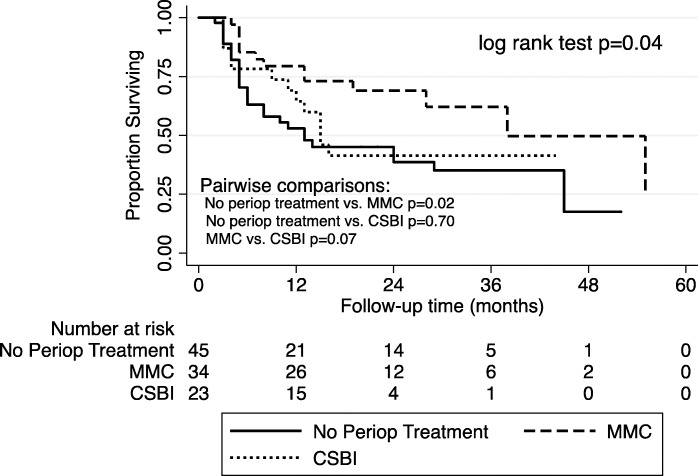
“DFS in Patients with High Risk”. Kaplan-Meier survival curve for patients with high risk disease stratified by perioperative treatment. MMC, Mitomycin-C. CSBI, continuous saline bladder irrigation

Median follow-up time for the entire cohort was 16 [Interquartile range (IQR): 8–28] months. A total of 74 (36.1%) patients recurred at a median of 9.5 [IQR: 4–14] months and 16 (7.8%) progressed at a median of 16 [IQR: 6–31.5] months. The median DFS was 25 months for those who received no perioperative treatment, 55 months for those receiving MMC, and 16 months for those receiving CSBI. The Kaplan-Meier survival curve is presented in Fig. 1 and demonstrates a significant DFS advantage of MMC compared with either CSBI or no perioperative treatment (log rank test: *p* < 0.01). Kaplan-Meier curves for patients with a combination of low and intermediate risk NMIBC (log rank test: *p* = 0.02) and high risk NMIBC (log rank test: *p* = 0.04), and are presented in Figs. 2 and 3, respectively.

Lastly, we created a multivariable model incorporating age, AUA risk stratification, use of additional adjuvant therapy, and type of perioperative therapy (None, MMC, or CSBI). On Cox multivariable modeling, high risk was associated with increased risk of recurrence or progression (HR 2.77, 95% CI: 1.28–6.01), whereas adjuvant therapy (HR 0.35, 95% CI: 0.20–0.59) and MMC (HR 0.43, 95% CI: 0.25–0.75) were associated with decreased risk of recurrence or progression (Table 3).

**Table 3:** Cox multivariable model for Recurrence or Progression

Variable	Hazard Ratio	95% Confidence Interval	p-value
Age (per year of age)	1.00	0.98–1.02	0.92
AUA Risk Stratification			
Low Risk	Reference	Reference	
Intermediate Risk	0.84	0.39–1.80	0.66
High Risk	2.77	1.28–6.01	0.01
Adjuvant therapy			
No	Reference	Reference	
Yes	0.35	0.20–0.59	< 0.001
Perioperative treatment			
No perioperative treatment	Reference	Reference	
MMC	0.43	0.25–0.75	0.003
CSBI	0.96	0.58–1.60	0.89

*LG* low grade, *HG* high grade, *CIS* carcinoma in situ, *MMC* Mitomycin-C, *CSBI* continuous saline bladder irrigation

## Discussion

The burden of NMIBC includes high financial costs to the healthcare system, significant risk of recurrence that necessitates life-long invasive surveillance, and uncertainty of possible progression that would prompt future radical operative intervention, especially in the highest-risk patients. Strategies to reduce the risk of recurrence and progression, including intravesical chemotherapy and immunotherapy, have been shown to be effective. [4, 12] However, none of these are without risk of potential significant side effects. In our current study we sought to utilize postoperative CSBI in a fashion similar to MMC, as an immediate, one-time postoperative treatment following surgery. This strategy avoids the toxicity of intravesical chemotherapy, as well as the inconvenience of an overnight hospital stay for prolonged CSBI.

In our cohort, however, post-operative CSBI for two hours was not equivalent to a single dose of perioperative MMC. Given the small numbers of patients in the low risk subgroup, we combined patients from low risk and intermediate risk groups for analysis. In the low and intermediate risk patients, there was a significant improvement in DFS with MMC compared with CSBI. In fact, CSBI performed no better than no perioperative treatment. In the high risk subgroup, a similar trend was observed. In our study the absolute risk reduction of postoperative MMC compared with no treatment at one year was 12.3%, which is similar to what is reported in the literature (11.7%). [4, 13] This benefit of MMC holds true even in our Cox multivariable model.

With respect to the efficacy of CSBI, our data stands in contrast to results published by others, albeit with some important differences in study design. Onishi et al. performed a non-randomized study comparing 18–22 h of post-operative CSBI to a full year of induction and maintenance MMC in patients with European Organization for Research and Treatment of Cancer (EORTC) intermediate risk NMIBC and showed no difference in several outcomes, including recurrence-free rates, time to first recurrence, and frequency of recurrences. [10] In this manuscript, the authors alluded to a planned prospective study that was recently published. [14] In their follow-up study, 227 patients with primary EORTC low- to intermediate-risk (all LG) NMIBC were randomized 1:1 to receive CSBI for 18 h or a single dose of 30 mg of MMC in 30 mL of saline. After a median follow-up of 37 months, 29% of patients experienced a recurrence. Recurrence-free rates at 1, 3, and 5 years were similar between the CSBI and MMC groups on Kaplan-Meier analysis. Subgroup analysis showed no difference when stratified between the low- and intermediate-risk tumors. Adverse events were also compared and the MMC group was found to have significantly higher rates of gross hematuria, irritative bladder symptoms, and dysuria (including retention). While the equivalence of CSBI and MMC demonstrated by Onishi et al. could be explained in part by patient selection (all LG patients), we did not replicate this result even in the low and intermediate risk subgroups of our cohort. One important difference in our protocols is the dose of MMC, which was the standard 40 mg in our study and 30 mg in the study by Onishi et al. The most striking difference between our studies, however, is in the duration of CSBI. We intentionally restricted CSBI to two hours to limit the need for overnight hospital stays. While similarly efficacious to one instillation of MMC, CSBI used by Onishi et al. was titrated over 18 h, and it was not reported how many of these patients required an overnight stay. While the authors debate the cost advantages of saline compared with MMC, we question whether this may be offset by even a small fraction of patients requiring overnight admissions for CSBI. Nevertheless, this data demonstrates that in addition to a standard dose of 40 mg of MMC, duration may be an important component of the efficacy of CSBI in preventing tumor cell re-implantation.

Our results also appear to conflict with the results of a recent meta-analysis utilizing individual patient data from randomized trials comparing immediate intravesical instillation of various chemotherapy agents to TURBT alone or instillation of control solution (saline or water). [5] Upon closer examination, however, we are unable to compare the protocols included as published in the meta-analysis or in the original manuscripts to our brief post-operative irrigation protocol. Of the 13 included studies, the use of post-operative irrigation was only documented as consistently used in four of these studies. Irrigation protocols were not detailed in the meta-analysis and review of the original data could not identify specific protocols. Furthermore, at least one study utilized distilled water for irrigation, which has the theoretical advantage of an osmotic cytotoxic effect but the disadvantages of being hypotonic. Therefore, despite a 21% relative reduction in recurrences found in this meta-analysis with use of post-operative irrigation alone, we can only cautiously compare this result with our data without more detailed information about the irrigation protocols used.

The concept of utilizing irrigation for eradication of residual tumor cells following surgery for cancer is not a new concept, nor is it limited to urology or even endoscopic surgery. Surgeons have traditionally irrigated surgical sites to mechanically wash away debris, dilution of bacterial loads, and as a method of tumor cell lysis, depending on the tonicity of the fluid. A survey in England found that 74% of general surgeons perform intraoperative peritoneal lavage during cancer operations (36% with water, 21% with saline, and 17% with betadine). [15] However, efficacy data on irrigation type is conflicting. Sweitzer et al. designed an experiment in mice to evaluate whether distilled water or sterile saline irrigation could reduce the burden of orthotopically implanted melanoma tumor cells. [16] Unfortunately, they found that neither the mechanical process of irrigation nor the hypotonicity of water reduced the tumor burden. In contrast, Fumito et al. demonstrated the superiority of water irrigation to saline irrigation following laparotomy in a mouse model of colorectal cancer tumor spillage. [17] In head and neck cancer models, both the type of irrigation and type of cancer cell line contributed to efficacy. [18, 19] These and other conflicting data suggest that multiple factors play a role with respect to the eradication of residual tumor burden, potentially related to the microenvironment and tumor cell-specific factors, such as cell adhesion properties and degree of de-differentiation.

The literature does strongly support irrigation following intra-luminal surgery in other surgical fields. For example, Zhou et al. performed a meta-analysis of studies evaluating intra-luminal washout following anterior resection for rectal cancer and concluded that washout leads to reduced rates of local recurrence. [20] In the urologic literature, Moskovitz et al. first postulated in 1987 that intravesical irrigation with distilled water during and after TURBT would lead to fewer recurrences. [21] While several small studies have demonstrated conflicting results regarding the use of water irrigation compared with no perioperative treatment, no studies have compared CSBI to MMC until the aforementioned studies by Onishi et al. [10, 14, 22, 23] Our study is the first to compare a shorter, perioperative duration of CSBI to both MMC and no perioperative treatment, and to evaluate this strategy in a heterogeneous patient population with low, intermediate, and high risk disease.

Our results, however, should be considered within the context of several limitations. Although this was a hypothesis-based study driven by pre-clinical and clinical data, it was not a randomized controlled study, and was limited to the data available in medical records. Furthermore, the study is underpowered and longer term follow-up is required to fully realize the potential differences between treatment groups. It is possible that a larger cohort with longer term follow up could confirm the null hypothesis, suggesting that no difference exists between treatment groups. However, at our institution we are mainly utilizing intravesical gemcitabine based on recently published data that suggests efficacy at a fraction of the cost and with reduced side effects compared with MMC. [24] Consequently, in combination with the current data that suggests inefficacy of 2 h of CSBI, we are unlikely to treat more patients with adjuvant CSBI. Primarily one surgeon (KC) performed CSBI during the study period while most other surgeons in the department utilized either MMC or no additional perioperative therapy. Therefore, referral patterns may have contributed to patient heterogeneity between groups. Despite some baseline differences between treatment groups described in our results, the data remains consistent when controlling for factors such as tumor grade, stage, and recurrence disease, among others, in a multivariable model. A consistent surveillance cystoscopy protocol was not used for all patients and could have helped standardize follow-up and limit detection bias. Finally, we utilized a clinical definition of recurrence that included any suspicious lesion during office cystoscopy that warranted an intervention (usually fulguration), which may have artificially increased our recurrence rates.

Nevertheless, our study comparing perioperative CSBI, perioperative MMC, and no perioperative treatment answers important questions regarding CSBI as prophylaxis following endoscopic resection for NMIBC. While CSBI for two hours postoperatively should not replace current guideline-recommended perioperative MMC, it does appear that longer duration of CSBI may increase its efficacy. [10, 14] Research is needed to determine whether the duration can be reduced to limit the number of additional hospital stays and whether other, novel perioperative instillations may reduce recurrences and limit side effects.

## Conclusions

Our data demonstrates that perioperative CSBI for two hours following TURBT is not equivalent to postoperative MMC in terms of rates of recurrence or progression. CSBI for two hours appears to be equivalent to no perioperative treatment, regardless of tumor grade. It is possible that CSBI may be required for a longer duration to reduce tumor cell re-implantation and, in turn, decrease rates of recurrence or progression.
